# Interplay between sex, age, BMI, health-related quality of life, and coping strategies in amateur and professional athletes

**DOI:** 10.3389/fphys.2025.1626037

**Published:** 2025-06-24

**Authors:** Walter Sapuppo, Davide Giacconi, Antonietta Monda, Antonietta Messina, Daniele Saccenti, Claudia Maria Mineo, Maria Casillo, Salvatore Allocca, Giovanni Michelini, Regina Gregori Grgič, Vincenzo Monda, Jacopo Lamanna, Mattia Ferro, Girolamo Di Maio, Marcellino Monda, Marco La Marra

**Affiliations:** ^1^ Department of Psychology, Sigmund Freud University Wien, Milan, Italy; ^2^ Studi Cognitivi, Cognitive Psychotherapy School and Research Center, Milan, Italy; ^3^ Department of Psychology, INSPIRE Lab, Sigmund Freud University Wien, Milan, Italy; ^4^ Department of Human Science and Quality of Life Promotion, San Raffaele Telematic University, Rome, Italy; ^5^ Department of Precision Medicine, University of Campania “Luigi Vanvitelli”, Naples, Italy; ^6^ Department of Experimental Medicine, University of Campania “Luigi Vanvitelli”, Naples, Italy; ^7^ Department of Disabilities, Fondazione Istituto Ospedaliero di Sospiro, Sospiro, Italy; ^8^ Department of Economics, Law, Cybersecurity, and Sports Sciences, University of Naples “Parthenope”, Naples, Italy; ^9^ Faculty of Psychology, Vita-Salute San Raffaele University, Milan, Italy; ^10^ Department of Psychology and Health Sciences, Pegaso Telematic University, Naples, Italy

**Keywords:** health-related quality of life, coping strategies, athletes, Body Mass Index, physical activity, amateur and professional sports

## Abstract

**Background/Objectives:**

The psychological well-being of athletes has garnered increasing interest due to its strong association with physical performance. While somatic indicators such as Body Mass Index (BMI) are routinely monitored in sports, the role of psychological resources—especially coping strategies—in shaping Health-Related Quality of Life (HRQoL) remains underexplored. This study aimed to investigate the influence of psychological and physical factors on HRQoL among amateur and professional athletes, controlling for sex, age, and BMI.

**Methods:**

A cross-sectional design was adopted, involving 537 athletes (326 males, 211 females; mean age = 32.44, SD = 13.64), aged 18–76 years. Participants were recruited via online platforms and sports organizations and completed a battery of self-report questionnaires, including the SF-36 to assess HRQoL and the COPE-NVI-25 to evaluate coping strategies. BMI was calculated from self-reported height and weight. Hierarchical multiple regression analyses were performed to assess the relative contributions of demographic, anthropometric, and psychological variables to HRQoL.

**Results:**

Demographic variables and BMI explained a limited proportion of the variance in HRQoL. In contrast, coping strategies significantly contributed to HRQoL outcomes, accounting for up to 22.5% of the variance in the global SF-36 score. Positive attitude and social support were associated with better physical and mental health, while avoidance strategies showed consistent negative associations across all HRQoL dimensions.

**Conclusion:**

Adaptive coping strategies, particularly positive attitude and social support, play a pivotal role in enhancing athletes’ HRQoL, surpassing the influence of BMI, sex, and age. These findings support the development of tailored psychological interventions to foster athlete wellbeing across competitive levels.

## 1 Introduction

The relationship between Health-Related Quality of Life (HRQoL), coping strategies, and physical indicators such as Body Mass Index (BMI) has been widely examined across various populations (Lerdal et al., 2011; Mason et al., 2017; Torrente-Sánchez et al., 2021; Janiczak et al., 2022; Moon and Han, 2022; Paunescu et al., 2024; Stefanovics et al., 2024). However, these dynamics remain insufficiently explored within athletic populations, despite the pivotal role of physical activity in promoting both physical and psychological well-being (Martinsen, 2000; Rebar et al., 2015; McKeon et al., 2022; Pearce et al., 2022; Mahindru et al., 2023; Singh et al., 2023; Sánchez-Alcalá et al., 2023; Limone et al., 2024; Liu C. et al., 2024). This aspect gains further relevance in light of studies highlighting the cognitive implications of excess weight, including alterations in executive functioning (La Marra et al., 2022c; La Marra et al., 2022d; La Marra et al., 2022a; Ilardi et al., 2024). Athletes, whether amateur or professional, constitute a heterogeneous group characterized by specific anthropometric features and physical demands that often differ substantially from those of the general population (Silva et al., 2013; Michalsik et al., 2015; Menargues-Ramírez et al., 2022; Pfeifer et al., 2022; Larkin et al., 2023). Physical activity is broadly recognized as a cornerstone of health promotion, contributing to enhanced cardiovascular function, metabolic efficiency, and mental health (Chieffi et al., 2017; Lavie et al., 2019; Schuch and Vancampfort, 2021). Nevertheless, its impact can vary significantly depending on the intensity, frequency, and type of sport practiced (Warburton, 2006; Poitras et al., 2016; Bull et al., 2020; Fyfe et al., 2022; Trajković et al., 2023; Jaekel, 2024). HRQoL, commonly assessed through validated instruments such as the SF-36 questionnaire, offers a multidimensional evaluation of health by encompassing both physical and psychological domains. These tools facilitate a comprehensive understanding of athletes’ health profiles by addressing not only somatic parameters but also emotional and cognitive dimensions essential for overall well-being (Geithner et al., 2006; Chieffi et al., 2014; Chieffi et al., 2019; Grosprêtre and Lepers, 2016; Scharfen and Memmert, 2019; Yongtawee et al., 2022; Staśkiewicz-Bartecka et al., 2023). The psychological dimensions of physical activity are particularly relevant in the athletic context. Regular engagement in sports fosters mental resilience, emotional regulation, and effective stress management. In this regard, coping strategies, defined as the cognitive and behavioral efforts employed to manage internal or external stressors, emerge as a fundamental determinant of psychological health (Sarkar and Fletcher, 2014; Dahlstrand et al., 2021; Forresi et al., 2022; Daley and Reardon, 2024; Liu M. et al., 2024; Sandi et al., 2024; Schinke et al., 2024). Adaptive coping mechanisms, such as problem-solving, positive reframing, and seeking social support, are positively associated with reduced psychological distress, improved emotional balance, and enhanced recovery processes (Schut and Schut, 1999; Bjørlykhaug et al., 2022). Conversely, maladaptive strategies, including avoidance, denial, or emotional suppression, have been linked to increased vulnerability to stress-related disorders and may adversely affect both health and performance (Ruggiero et al., 2017; McNeil et al., 2024; Miller et al., 2024; Rogers et al., 2024; Sapuppo et al., 2024c; Dišlere et al., 2025). Furthermore, psychological well-being is closely intertwined with an athlete’s ability to cope with the pressures of competition, recover from setbacks, and remain committed over time (Büttner et al., 2021; Martín-Rodríguez et al., 2024; Sanz-Matesanz et al., 2024; Sapuppo et al., 2024a). Physical activity has also been shown to alleviate symptoms of anxiety and depression, enhance mood states, and support cognitive function, contributing to greater self-efficacy and overall life satisfaction (Dalkiliç, 2017; Krenn et al., 2018; Scharfen and Memmert, 2019; Ilardi et al., 2020; Yongtawee et al., 2022; Logan et al., 2023). For athletes, fostering a robust psychological foundation is essential not only for optimizing performance but also for promoting long-term health and personal development. Mental and emotional well-being represent integral components of a sustainable and healthy lifestyle (Chang et al., 2020; Varghese et al., 2022; Eather et al., 2023). A critical aspect in this context is the observed disparity in quality of life between athletes and non-athletes. Generally, athletes report higher levels of physical well-being due to the physiological benefits of regular exercise, such as improved cardiovascular capacity, muscular strength, and aerobic fitness. However, those engaged in high-intensity or professional sports are also exposed to specific risks, including chronic injuries, overuse syndromes, and elevated psychological stress, which can detract from their overall quality of life (Kucharski et al., 2018; Penttilä et al., 2022; Rose et al., 2023; Taheri et al., 2023; Sapuppo et al., 2024b). In contrast, non-athletes may be less exposed to sport-related risks but are often more susceptible to chronic conditions linked to sedentary behaviors, such as obesity, type 2 diabetes, and cardiovascular disease (Arocha Rodulfo, 2019; Lautenbach et al., 2021; Fruchart and Rulence-Pâques, 2022; Kim and Choi, 2022). This duality underscores the necessity for a nuanced understanding of quality of life, one that considers the interplay between physical, psychological, and contextual factors. The present study aims to investigate the relationships among HRQoL and coping strategies in a cohort of athletes, comprising both amateur and professional individuals. To ensure a comprehensive analysis, sex, age, and BMI were included as control variables, given their potential influence on health-related outcomes. By addressing these elements, the study seeks to provide an integrated perspective on the health status of athletes and the complex interrelations among their physical, psychological, and social needs. As physical activity continues to be emphasized as a public health priority, understanding the specific challenges and protective factors in athletic populations can offer valuable insights not only for sports science but also for broader health promotion strategies.

## 2 Methods

### 2.1 Participants

For a deeper understanding of the topic presented a cross-sectional study was conducted. Participants were recruited from the general population and through contact with some professional and semi-professional teams and clubs on a totally voluntary basis. Subjects were recruited through online platforms (e.g., social media, e-mail), and the whole battery of questionnaires was administered online. In the following study, adult subjects (i.e., aged 18 years old or more) taking part in sport at a competitive or amateur level, or having done so in the past, were considered. This research was conducted on a group of 537 athletes (amateur and professional). Moreover, inclusion criteria required participants to be actively engaged in training and competition for at least 1 year prior to the study. The current study is part of a larger research aimed at assessing cognitive characteristics, coping strategies, general health and well-being and supplements consumption in athletes.

### 2.2 Instruments

The study was conducted using the following instruments:

1. Health-Related Quality of Life (HRQoL): Assessed using the 36-Item Short Form Survey (SF-36) which evaluates physical and mental health domains. The questionnaire consists of 36 item that can be considered as a single scale (global score) or divided into 8 subscales: physical functioning (PF) (10 items), limitations due to physical health (Role Physical - RP) (4 items) limitations due to Emotional issues (Role Emotional - RE) (3 items), energy and dissatisfaction (Vitality - VT) (4 items), psychological well-being (Mental Health - MH) (5 items), social engagement (Social Functioning - SF) (2 items), pain (Bodily Pain - BP) (2 items), and general health perception (General Health - GH) (5 items). Higher scores indicate better quality of life (Apolone and Mosconi, 1998).
2. Coping strategies (Coping Orientation to Problems Experienced–New Italian version; COPE-NVI-25): This is a 25-item self-report questionnaire divided into 5 scales, each consisting of 2 items. The questionnaire is scored from 1 to 4, ranging from “Not at all” to “Very much”, and is designed to measure effective and ineffective coping mechanisms for stressful life events. Every scale is examined independently: (1) avoidance strategies, (2) transcendent orientation, (3) positive attitude, (4) social support, (5) problem orientation. Higher scores indicate a more persistent use of the strategies assessed (Caricati et al., 2015).
3.24-item ad hoc self-report survey: For this cross-sectional study, a 24-item questionnaire was specifically developed to collect participants descriptives data on the following domains: (1) level of physical activity, (2) type of sport practiced, and (3) frequency and duration of sport participation. Additionally, basic demographic and anthropometric information, such as nationality, age, gender, weight, and height, was collected to allow for a comprehensive characterization of the study population. Additionally, the data gathered were used to measure participants BMI, calculated as weight (kg) divided by height squared (m^2^). It should be noted that the questionnaire was not based on a previously validated or standardized instrument. Rather, it was designed ad hoc to address the specific objectives of the study and to gather targeted information relevant to the research questions.

### 2.3 Study design and procedure

This is a non-clinical cross-sectional study conducted between June 2024 and March 2025. Subjects were recruited through online, sport clubs and gyms and the questionnaire were completed digitally through the platform “Google Forms”. Before completing the questionnaire, subjects were asked to give their consent to participate in the study by agreeing to an informed consent regarding data processing for scientific and research purposes. Participants were informed about the purpose of the study and anonymity of the data collection and analysis.

### 2.4 Statistical analysis

First, descriptive statistics were computed on the data. Then, one-sample t-tests were conducted to compare the mean values obtained from our sample with those derived from the general population regarding health-related quality of life domains and coping strategies. Chi-squared tests of independence were used to assess the association between demographical and physical-activity related dimensions. Nine hierarchical multiple regression analyses were also conducted. Regression provided an understanding of how much variance in health-related quality of life could be accounted for by coping strategies and allowed to examine patterns and determine which strategies are significant, unique predictors of different domains of health-related quality of life. One regression predicted the global SF-36 score as the criterion variable, and the remaining eight regressions each predicted either the role physical, role emotional, social functioning, physical functioning, vitality, bodily pain, general health or mental health subscale scores of the SF-36. Demographic variables (i.e., age and gender) as well as BMI were entered in the first block of predictors to control for their potential effect as covariates. All five of the COPE-NVI-25 subscales scores were entered in the second block of predictors. Normality was evaluated using the Shapiro–Wilk test and visual inspection of Q-Q plots. The statistical significance cut-off level was set at *p* < 0.05, 2-tailed. Data analysis was conducted utilizing statistical software, specifically IBM SPSS Statistics (Version 29.0.2.0).

### 2.5 Ethics

The study, along with the questionnaires used and the methods of data collection and storage, was approved by the Ethics Committee of Sigmund Freud University, Ethics Commission of the Faculty of Psychotherapy Science and the Faculty of Psychology; BD5VKJDAC4UJIC91006; 30 July 2024. The reference for this approval is GCP4Q7JFBO3P6I90070.

## 3 Results

### 3.1 Descriptive statistics

A total of 537 athletes (326 males and 211 females) participated in the survey. The vast majority of them were Italian (98.4%), while the remaining (1.6%) reported to come from France (n = 2; 0.4%), Switzerland (n = 2; 0.4%), India (n = 1; 0.2%), Peru (n = 1; 0.2%), Russia (n = 1; 0.2%), and Slovenia (n = 1; 0.2%). The mean age of the sample was 32.44 (±13.64) years, ranging from 18 to 76 years of age. The mean BMI recorded within the sample was 24.22 (±4.07) kg/m^2^, ranging from a minimum of 15.21 kg/m^2^ to a maximum of 42.45 kg/m^2^. Health-related quality of life scores extracted from the SF-36, as well as the coping strategies investigated through the COPE-NVI-25 are outlined in Table 1.

**TABLE 1:** Summary of the health-related quality of life scores and coping strategies of the athletes.

Variable	Mean	Standard Deviation	Range	α	Mean^a^	Standard Deviation^a^
SF-36						
Role Physical	81.19	29.73	0.00–100	0.77	78.21	35.93
Role Emotional	69.96	38.30	0.00–100	0.79	76.16	37.25
Social Functioning	64.34	24.67	0.00–100	0.41	77.43	23.34
Physical Functioning	94.55	10.43	25.0–100	0.82	84.46	23.18
Bodily Pain	82.25	19.93	10.0–100	0.79	73.27	27.65
General Health	66.41	15.05	20.0–100	0.60	65.22	22.18
Vitality	55.76	15.50	5.00–100	0.71	61.89	20.69
Mental Health	63.90	16.64	5.00–100	0.82	66.59	20.89
COPE-NVI-25						
Avoidance Strategies	2.28	0.82	1.00–5.80	0.68	2.43	1.02
Transcendent Orientation	1.61	1.14	1.00–6.00	0.97	2.7	1.61
Positive Attitude	4.30	0.98	1.17–6.00	0.86	4.46	0.94
Social Support	3.74	1.24	1.00–6.00	0.91	3.67	1.2
Problem Orientation	3.91	0.91	1.00–6.00	0.79	4.46	0.94

^a^ Values in general population extracted from (Apolone and Mosconi, 1998; Caricati et al., 2015).

One sample t-tests highlighted statistically significant differences between our sample and the mean scores retrieved from the general population in the following health-related quality of life domains: *role physical* (*t*(536) = 2.32, *p* < 0.05, Cohen’s *d* = 0.10), *role emotional* (*t*(536) = 3.75, *p* < 0.001, Cohen’s *d* = 0.25), *social functioning* (*t*(536) = 12.30, *p* < 0.001, Cohen’s *d* = 0.53), *physical functioning* (*t*(536) = 22.43, *p* < 0.001, Cohen’s *d* = 0.97), *bodily pain* (*t*(536) = 10.44, *p* < 0.001, Cohen’s *d* = 0.45), vitality (*t* (536) = 9.16, *p* < 0.001, Cohen’s *d* = 0.40), and *mental health* (*t*(536) = 3.74, *p* < 0.001, Cohen’s *d* = 0.16). Moreover, statistically significant differences between our sample and the mean scores extracted from the general population were detected in most coping strategies, including *avoidance* (*t*(536) = 4.20, *p* < 0.001, Cohen’s *d* = 0.18), *transcendent orientation* (*t*(536) = 22.01, *p* < 0.001, Cohen’s *d* = 0.95), *positive attitude* (*t*(536) = 3.77, *p* < 0.001, Cohen’s *d* = 0.16), and *problem orientation* (*t*(536) = 13.92, *p* < 0.001, Cohen’s *d* = 0.60).

In the remaining part of the assessment, participants reported having practiced regular physical activity for a mean of 16.71 (±12.08) years, ranging from a minimum of 1 to a maximum of 60 years. Additional descriptive statistics on more specific facets of physical activity were calculated, including the regularity of physical activity, the prevailing motivation behind physical effort, and the competitive or professional level at which physical activity was practiced. Frequencies were broken down by gender and summarized in Table 2.

**TABLE 2:** Self-report summary of physical activity-related specifications.

Variable	Males	Females	Total
Physical activity regularity			
My physical activity has been generally constant over the years	220 (40.97%)	117 (21.79%)	337 (62.76%)
I used to practice more physical activity than I do now	83 (15.46%)	72 (13.41%)	155 (28.86%)
I used to practice less physical activity than I do now	23 (4.28%)	22 (4.09%)	45 (8.38%)
Prevailing motivation behind practicing physical activity			
Competition	32 (5.96%)	12 (2.23%)	44 (8.19%)
Entertainment and social occasions	37 (6.89%)	18 (3.35%)	55 (10.24%)
Improvement of body image	17 (3.17%)	36 (6.70%)	53 (9.87%)
Improvement of physical and psychological health	208 (38.73%)	133 (24.77%)	341 (79.89%)
Improvement of athletic performance	31 (5.77%)	13 (2.43%)	44 (8.19%)
Agonistic level physical activity			
I’ve never competed at the agonistic level	44 (8.19%)	43 (8.01%)	87 (16.20%)
Not currently, but I have competed at the agonistic level in the past	169 (31.47%)	71 (13.22%)	240 (44.69%)
I currently compete at the agonistic level	113 (21.04%)	97 (18.07%)	210 (39.11%)
Professional level physical activity			
I’ve never competed at the professional level	306 (56.98%)	109 (29.30%)	496 (92.36%)
Not currently, but I competed at the professional level in the past	16 (2.98%)	21 (3.91%)	37 (6.89%)
I currently compete at the professional level	4 (6.83%)	0 (0.0%)	4 (0.75%)

Chi-squared tests of independence showed significant associations between sex and the following physical activity-related domains: physical activity regularity (χ^2^(2) = 8.02, *p* < 0.05), prevailing motivation behind practicing physical activity (χ^2^(4) = 24.16, *p* < 0.001), agonist level physical activity (χ^2^(2) = 17.42, *p* < 0.001), and professional level physical activity (χ^2^(2) = 7.52, *p* < 0.05).

Subjects reported to be engaged in different athletic disciplines, including: Fitness activities (29.5%), Rugby (19.9%), Soccer (9.8%), Basketball (8.7%), Martial Arts (5.4%), Volleyball (4.9%), Swimming (4.5%), Cycling (3.4%), Tennis (2.0%), Gymnastics (1.9%), Breakdance (1.7%), Track and Field (1.7%), Skiing (1.5%), Rowing (1.1%), Skating (0.9%), Horse Riding (0.7%), Climbing (0.6%), Padel (0.4%), Table Tennis (0.4%), Badminton (0.2%), Baseball (0.2%), Hockey (0.2%), Water Polo (0.2%), and Target Shooting (0.2%). Those who participated in the above-mentioned athletic disciplines had practiced so for an average of 12.27 years (±10.16), ranging from 1 to 55 years.

### 3.2 Interplay among BMI, coping strategies, and health-related quality of life

Hierarchical multiple regression analyses were conducted to examine the linear relationships among athletes’ demographic characteristics (i.e., age and sex), BMI, their commonly employed coping strategies, and their levels of health-related quality of life.

#### 3.2.1 Global score

At step one, age, sex, and BMI accounted for a significant 6.4% of the variance in health-related quality of life. At step two, coping strategies accounted for a significant, additional, 22.5% of the variance (Δ*R* ^2^ = 0.16, Δ*F* (5, 527) = 21.90, *p* < 0.001), indicating medium effect size (*f* ^2^ = 0.29). Besides age (*β* = 0.19, *p* < 0.001), two coping strategies were significant unique predictors (see Table 3). Namely, heightened employment of *positive attitude* was associated with higher levels of health-related quality of life (*β* = 0.10, *p* < 0.05). Conversely, heightened recourse to *avoidance* was associated with lower levels of health-related quality of life (*β* = −0.35, *p* < 0.001).

**TABLE 3:** Summary of the hierarchical multiple regressions.

	Global Score			General Health			Bodily Pain		
	*t*-value	Standardized *β*	*p*-value	*t*-value	Standardized *β*	*p*-value	*t*-value	Standardized *β*	*p*-value
Step 1									
Age	5.29	0.24	<0.001	0.27	0.01	0.79	3.33	0.15	<0.001
Gender	−1.72	−0.08	0.09	−0.62	−0.03	0.54	0.31	0.01	0.76
Body Mass Index	−1.08	−0.05	0.28	−0.01	0.00	0.99	−3.49	−0.17	<0.001
Step 2									
Age	4.30	0.19	<0.001	−0.41	−0.02	0.68	3.07	0.15	<0.01
Gender	−1.78	−0.08	0.08	−0.90	−0.04	0.37	0.16	0.01	0.88
Body Mass Index	−0.32	−0.01	0.75	0.59	0.03	0.56	−3.25	−0.16	<0.01
Avoidance Strategies	−8.80	−0.35	<0.001	−5.51	−0.24	<0.001	−4.57	−0.20	<0.001
Transcendent Orientation	−0.85	−0.03	0.40	−0.69	−0.03	0.49	−0.32	−0.01	0.75
Positive Attitude	2.30	0.10	<0.05	1.86	0.09	0.06	1.93	0.09	0.06
Social Support	1.51	0.07	0.13	2.26	0.11	<0.05	1.96	0.10	<0.05
Problem Orientation	0.37	0.02	0.71	0.80	0.04	0.43	−1.64	−0.09	0.10

	Social Functioning			Mental Health			Vitality		
	*t*-value	Standardized *β*	*p*-value	*t*-value	Standardized *β*	*p*-value	*t*-value	Standardized *β*	*p*-value
Step 1									
Age	6.20	0.28	<0.001	3.70	0.17	<0.001	4.68	0.21	<0.001
Gender	−1.47	−0.07	0.14	−3.59	−0.17	<0.001	−2.42	−0.11	<0.05
Body Mass Index	−0.62	−0.03	0.54	−0.91	−0.04	0.36	−0.24	−0.01	0.81
Step 2									
Age	5.19	0.24	<0.001	2.69	0.12	<0.01	3.28	0.15	<0.01
Gender	−1.32	−0.06	0.19	−3.83	−0.17	<0.001	−2.58	−0.12	<0.05
Body Mass Index	−0.08	0.00	0.93	−0.33	−0.02	0.75	0.44	0.02	0.66
Avoidance Strategies	−4.72	−0.20	<0.001	−7.00	−0.29	<0.001	−7.55	−0.31	<0.001
Transcendent Orientation	−0.96	−0.04	0.37	−0.25	−0.01	0.80	0.63	0.03	0.53
Positive Attitude	1.92	0.10	<0.05	3.40	0.16	<0.001	2.47	0.11	<0.05
Social Support	0.13	0.01	0.90	1.36	0.06	0.17	0.77	0.04	0.44
Problem Orientation	0.83	0.04	0.41	−0.14	−0.01	0.89	1.05	0.05	0.30

	Role Emotional			Role Physical			Physical Functioning		
	*t*-value	Standardized *β*	*p*-value	*t*-value	Standardized *β*	*p*-value	*t*-value	Standardized *β*	*p*-value
Step 1									
Age	4.57	0.21	<0.001	2.00	0.09	<0.05	−0.62	−0.03	0.54
Gender	−1.57	−0.07	0.12	0.13	0.01	0.90	0.19	0.01	0.85
Body Mass Index	0.32	0.02	0.75	−0.43	−0.02	0.67	−1.28	−0.06	0.20
Step 2									
Age	3.36	0.16	<0.001	1.76	0.09	0.08	−0.78	−0.04	0.44
Gender	−1.27	−0.06	0.20	0.14	0.01	0.89	0.06	0.00	0.95
Body Mass Index	0.86	0.04	0.39	−0.03	0.00	0.97	−0.92	−0.05	0.36
Avoidance Strategies	−5.76	−0.25	<0.001	−4.36	−0.19	<0.001	−4.50	−0.20	<0.001
Transcendent Orientation	0.26	0.01	0.80	−1.56	−0.07	0.12	−1.41	−0.06	0.16
Positive Attitude	−0.57	−0.03	0.57	0.43	0.02	0.66	4.05	0.20	<0.001
Social Support	−0.60	−0.03	0.55	1.56	0.08	0.12	2.05	0.10	<0.05
Problem Orientation	1.21	0.06	0.23	−0.04	0.00	0.97	−1.79	−0.09	0.08

t-value, t statistic from regression analysis; Standardized β, standardized beta coefficient; p-value, significance level. Step 1, regression model including only sociodemographic and anthropometric variables. Step 2, regression model including sociodemographic, anthropometric, and psychological variables.

#### 3.2.2 General health

At step one, age, sex, and BMI accounted for a non-significant 0.1% of the variance in *general health*. At step two, coping strategies accounted for a significant, additional, 10.2% of the variance (Δ*R* ^2^ = 0.10, Δ*F* (5, 527) = 11.89, *p* < 0.001), indicating a small effect size (*f* ^2^ = 0.11). Two coping strategies were significant unique predictors (see Table 3). Specifically, heightened reliance on *social support* was associated with higher levels of *general health (GH)* (*β* = 0.11, *p* < 0.05). On the contrary, heightened recourse to *avoidance* was associated with lower levels of *general health (GH)* (*β* = −0.24, *p* < 0.001).

#### 3.2.3 Bodily pain

At step one, age, sex, and BMI accounted for a significant 3.0% of the variance in *bodily pain (BP).* At step two, coping strategies accounted for a significant, additional, 8.8% of the variance (Δ*R* ^2^ = 0.05, Δ*F* (5, 527) = 6.16, *p* < 0.001), indicating a small effect size (*f* ^2^ = 0.09). Besides age (*β* = 0.15, *p* < 0.01) and BMI (*β* = −0.16, *p* < 0.01), three comping strategies were significant unique predictors (see Table 3). Namely, heightened employment of *social support* (*β* = 0.10, *p* < 0.05) and *positive attitude* (*β* = 0.10, *p* < 0.05) were associated with ameliorated *bodily pain (BP).* Conversely, heightened reliance on *avoidance* was associated with worsened *bodily pain (BP)* (*β* = −0.20, *p* < 0.001).

#### 3.2.4 Social functioning

At step one, age, sex, and BMI accounted for a significant 8.5% of the variance in *social functioning (SF)*. At step two, coping strategies accounted for a significant, additional, 14.9% of the variance (Δ*R* ^2^ = 0.06, Δ*F* (5, 527) = 7.86, *p* < 0.001), indicating a medium effect size (*f* ^2^ = 0.18). Besides age (*β* = 0.24, *p* < 0.001), two coping strategies were significant unique predictors (see Table 3). Indeed, heightened employment of *positive attitude* was associated with higher levels of *social functioning* (*β* = 0.10, *p* < 0.05). On the contrary, heightened recourse to *avoidance* was associated with lower levels of *social functioning* (*β* = −0.20, *p* < 0.001).

#### 3.2.5 Mental health

At step one, age, sex, and BMI accounted for a significant 6.2% of the variance in *mental health (MH)*. At step two, coping strategies accounted for a significant, additional, 18.8% of the variance (Δ*R* ^2^ = 0.13, Δ*F* (5, 527) = 16.47, *p* < 0.001), indicating a medium effect size (*f* ^2^ = 0.23). Besides age (*β* = 0.12, *p* < 0.01) and sex (*β* = −0.17, *p* < 0.001), two coping strategies were significant unique predictors (see Table 3). Namely, heightened reliance on *positive attitude* was associated with higher levels of *mental health* (*β* = 0.16, *p* < 0.001). Conversely, heightened recourse to *avoidance* were associated with lower levels of *mental health* (*β* = −0.29, *p* < 0.001).

#### 3.2.6 Vitality

At step one, age, sex, and BMI accounted for a significant 6.8% of the variance in *vitality (VT)*. At step two, coping strategies accounted for a significant, additional, 20.4% of the variance (Δ*R* ^2^ = 0.14, Δ*F* (5, 527) = 18.08, *p* < 0.001), indicating a medium effect size (*f* ^2^ = 0.26). Besides age (*β* = 0.15, *p* < 0.01) and sex (*β* = −0.12, *p* < 0.05), two coping strategies were significant unique predictors (see Table 3). Indeed, heightened employment of *positive attitude* was associated with improved *vitality* (*β* = 0.11, *p* < 0.05). On the contrary, heightened reliance on *avoidance* was associated with worsened *vitality* (*β* = −0.31, *p* < 0.001).

#### 3.2.7 Role emotional

At step one, age, sex, and BMI accounted for a significant 6.0% of the variance in *role emotional* (RE). At step two, coping strategies accounted for a significant, additional, 12.6% of the variance (Δ*R* ^2^ = 0.07, Δ*F* (5, 527) = 7.89, *p* < 0.001), indicating a small effect size (*f* ^2^ = 0.14). Besides age (*β* = 0.16, *p* < 0.001), one coping strategy was a significant unique predictor (see Table 3). Accordingly, heightened employment of *avoidance* was associated with worsened *role emotional* (*β* = −0.25, *p* < 0.001).

#### 3.2.8 Role physical

At step one, age, sex, and BMI accounted for a non-significant 0.8% of the variance in *role physical* (RP). At step two, coping strategies accounted for a significant, additional, 5.8% of the variance (Δ*R* ^2^ = 0.05, Δ*F* (5, 527) = 5.63, *p* < 0.001), indicating a small effect size (*f* ^2^ = 0.06). One out of the five coping strategies was a significant unique predictor (see Table 3), i.e., heightened recourse to avoidance was associated with worsened *role physical* (*β* = −0.19, *p* < 0.001).

#### 3.2.9 Physical functioning

At step one, age, sex, and BMI accounted for a non-significant 0.7% of the variance in *physical functioning* (PF). At step two, coping strategies accounted for a significant, additional, 9.3% of the variance (Δ*R* ^2^ = 0.09, Δ*F* (5, 527) = 9.95, *p* < 0.001), indicating a small effect size (*f* ^2^ = 0.10). Three out of the five coping strategies were significant unique predictors (see Table 3). Specifically, heightened reliance on *social support* (*β* = 0.10, *p* < 0.05) and *positive attitude* (*β* = 0.20, *p* < 0.001) were associated with higher levels of *physical functioning*. Conversely, heightened employment of *avoidance* was associated with diminished *physical functioning* (*β* = −0.20, *p* < 0.001).

## 4 Discussion

Our analyses investigated the impact of age, sex, BMI, and coping methods on diverse health-related quality of life (HRQoL) outcomes across athletes from multiple disciplines and competitive levels. While age, sex, and BMI contributed minimally to the variance in HRQoL (between 0.1% and 8.5%), coping techniques proved to be the most significant predictors, explaining up to 22.5% of the variance. These findings highlight the importance of psychological coping strategies in influencing subjective health and well-being in athletes. Among the adaptive strategies, both a *positive attitude* and reliance on *social support* emerged as key protective factors across several HRQoL domains. These strategies likely facilitate emotional regulation, injury recovery, and adherence to training routines, highlighting their relevance for psychological resilience in sports contexts (Murray et al., 2019; Budimir et al., 2021; Huang et al., 2021; Prior et al., 2024). Additionally, these results underline the importance of social connections, including those with coaches, teammates, family, or peers, for emotional processing, stress management, and compliance with training and recuperation regimens. Moreover, access to supportive connections can provide affirmation, diminish feelings of isolation, and strengthen adaptive actions (Graber et al., 2016; Sbrizzi and Sapuppo, 2021). In structured team settings or among professionals, the pursuit of social support may significantly influence both well-being and performance stability (Uchino, 2006; Koelmel et al., 2017; Hadebe and Ramukumba, 2020; Koch and Krenn, 2021; Wong et al., 2024). Conversely, avoidance coping consistently predicted poorer outcomes across physical and psychological domains, likely due to reduced emotional processing and help-seeking behaviors, which can foster chronic stress and undermine recovery (Nippert and Smith, 2008; Bányai et al., 2021; McLoughlin et al., 2024). These findings, in line with previous research indicating that coping techniques significantly influence the impact of stress on HRQoL in sports (Budimir et al., 2021; Fullerton et al., 2021; Huang et al., 2021; Prior et al., 2024). The disparities in stress management between professional and amateur athletes indicate that structured mental health support and coping mechanisms might alleviate stress in competitive contexts. Professional athletes may gain from structured mental health resources, while amateurs are more susceptible to maladaptive coping mechanisms, adversely impacting HRQoL (Jacobson and Matthaeus, 2014; Wong et al., 2024). Moreover, gender disparities were observed in the *mental health* and *vitality* subscales of the SF-36. Our findings indicate that female athletes exhibited diminished *mental health* scores, potentially due to increased susceptibility to psychological distress or sociocultural pressures concerning body image and performance expectations (Gattino et al., 2015; Tomaszek and Muchacka-Cymerman, 2019; Toselli et al., 2022). Conversely, men indicated elevated *vitality* scores, potentially attributable to variations in fatigue perception or recovery methodologies. Unlike in general population trends, BMI did not significantly predict most HRQoL outcomes in our sample, likely due to sport-specific physical profiles and the moderating role of coping strategies.

Although BMI was first employed as a covariate, our results indicate that it does not exhibit a unidirectional connection with HRQoL in athletes. Unlike in general population trends, BMI did not significantly predict most HRQoL outcomes in our sample, likely due to sport-specific physical profiles and the moderating role of coping strategies (Hopman et al., 2007; Milanović et al., 2022; Knettel et al., 2023; Rose et al., 2023). In fact, regression analysis indicated that BMI was not a significant predictor of *general health* or *social functioning*, but it exhibited a negative correlation with *bodily pain*. These findings indicate that a higher BMI may be associated with musculoskeletal strains without necessarily affecting the perception of overall health (Crewther et al., 2012; Martin and Beckham, 2020; Ruscello et al., 2024). Athletes participating in strength or contact sports often have high BMI values, which can improve physical function and performance (Harty et al., 2021; Van Baak et al., 2021; Entwistle et al., 2022; University of Girona, University School of Health and Sport, Girona, Spain et al., 2022; Baceviciene et al., 2023; Borowiec et al., 2023; Habay et al., 2023; Berengüí et al., 2024). Results obtained in this study suggest that a higher BMI does not necessarily correlate with diminished HRQoL in athletes, especially when considering their coping mechanisms and the nature of their sport. However, it is important to note that this finding should not be interpreted as evidence of an “obesity paradox” in the athletic population. In fact, in this context, the term may be misleading, as BMI does not accurately reflect body composition and cannot be reliably used to diagnose obesity in athletes. Instead, higher BMI values in athletes often reflect increased lean mass rather than excess fat (Walsh et al., 2018; Afzal et al., 2021; Villano et al., 2021b; Quesada et al., 2022; Monda et al., 2024; Monda et al., 2017; Simati et al., 2023; Zwartkruis et al., 2023; La Marra et al., 2024; Sparks et al., 2024; Yang et al., 2024; Zhao et al., 2025; Banack and Stokes, 2017; Childers and Allison, 2010; Simati et al., 2023). Body weight perceptions and self-acceptance fluctuate according to sports culture and role expectations (Koc, 2017; Paixão et al., 2021; Villano et al., 2021a; Ruiz-Turrero et al., 2022; Ahsan and Ali, 2023; Gao et al., 2023; Krupa-Kotara et al., 2023; Martín-Talavera et al., 2023; Zaccagni and Gualdi-Russo, 2023). Moreover, these findings demonstrated that the motivations for participating in sports are also significant. Most athletes in our sample indicated health-related motivations (79.89%), which exhibited a positive correlation with adaptive coping and HRQoL (Orbach et al., 2021; Aznar-Ballesta et al., 2022; Villano et al., 2022; Nuetzel, 2023). In contrast, athletes influenced by social or aesthetic expectations were more susceptible to maladaptive coping strategies, especially *avoidance*, resulting in diminished well-being (Bányai et al., 2021; McLoughlin et al., 2024). These trends emphasize the importance of fostering a sports culture that promotes health and psychological well-being rather than appearance or performance metrics (Sheehan et al., 2018; González et al., 2019; Koch and Krenn, 2021; Logan et al., 2023). Finally, emphasis must be placed on the post-athletic transition phase, wherein elevated BMI and diminished HRQoL have been commonly reported (Kelly et al., 2014; Buckley et al., 2019; Filbay et al., 2019; Silva et al., 2022; Le Roux et al., 2023; Street et al., 2023). Psychological challenges, such as body dissatisfaction and depressive symptoms, frequently arise during this phase due to alterations in identity and lifestyle (Iavarone, 2015; La Marra et al., 2022b; Furie et al., 2023; Pena-Pérez and Portela-Pino, 2023; Fatt et al., 2024; Runacres and Marshall, 2024). Researchers have suggested therapies that integrate physical activity, psychological counseling, and nutritional support to tackle these issues (Michaels et al., 2023; Voorheis et al., 2023; Vasileva et al., 2022; Claussen et al., 2024; Reinebo et al., 2024). Our data indicates that *social support* and a *positive attitude* continued to provide protection post-athletic retirement, so underscoring the enduring advantages of adaptive coping. Fostering a *positive attitude* and *social support* while reducing *avoidance* strategies may improve mental and physical health across all phases of an athletic career. These results could indicate the importance of creating athlete-centered preventative programs and establish a foundation for longitudinal studies examining the correlation between coping techniques and HRQoL.

### 4.1 Limitations

This study presents several limitations that should be acknowledged. Firstly, the cross-sectional nature of the design prevents causal inferences regarding the relationship between coping strategies and HRQoL. Longitudinal studies are needed to examine how coping mechanisms evolve over time and influence health outcomes throughout different stages of an athletic career. Secondly, the reliance on self-reported data may introduce potential biases, including recall inaccuracies and social desirability effects. This concern is particularly relevant for anthropometric variables, such as self-reported height and weight used to calculate BMI, as well as for coping strategies, which may reflect aspirational or socially acceptable responses rather than actual behavioral patterns. Future studies would benefit from incorporating objective measures (e.g., direct anthropometric assessments) and qualitative approaches to validate and complement self-reported data, thereby enhancing the reliability and ecological validity of the findings. Thirdly, although the sample included both amateur and professional athletes across a wide age range, it was not stratified by sport type, training intensity, or career stage, all of which may differentially impact coping and HRQoL. Furthermore, cultural, psychological, and socio-economic variables were not considered, limiting the generalizability of the findings to broader populations. Lastly, although the COPE-NVI-25 is a validated tool, the complex and dynamic nature of coping could benefit from a mixed-methods approach, integrating qualitative data to better capture individual experiences.

## 5 Conclusion

The present study highlights the central role of coping strategies, particularly positive attitude and social support in enhancing health-related quality of life among athletes. These adaptive mechanisms demonstrated a stronger association with HRQoL outcomes than physical indicators such as BMI, age, or sex. In contrast, the use of avoidance strategies was consistently linked to poorer physical and psychological health. These findings underscore the importance of promoting athlete-centered mental health interventions that encourage positive coping and reduce maladaptive behaviors. Practical implications include the integration of early psychological screening protocols within training settings to identify athletes at risk of maladaptive coping. Furthermore, tailored interventions, led by sports psychologists in collaboration with coaching staff, can foster the development of adaptive coping skills, such as positive reframing and effective help-seeking. Such efforts may contribute to enhanced well-being, injury recovery, and long-term engagement in sport. Future longitudinal and multi-method research is recommended to better elucidate the temporal dynamics of coping and to guide the implementation of evidence-based psychological support programs across all stages of an athletic career.

## Data Availability

The raw data supporting the conclusions of this article will be made available by the authors, without undue reservation.

## References

[B1] Afzal M. Siddiqi N. Ahmad B. Afsheen N. Aslam F. Ali A. (2021). Prevalence of overweight and obesity in people with severe mental illness: systematic review and meta-analysis. Front. Endocrinol. 12, 769309. 10.3389/fendo.2021.769309 PMC8656226 34899604

[B2] Ahsan M. Ali M. F. (2023). Body mass index: a determinant of distress, depression, self-esteem, and satisfaction with life amongst recreational athletes from random intermittent dynamic type sports. Heliyon 9, e15563. 10.1016/j.heliyon.2023.e15563 37128310 PMC10148114

[B3] Apolone G. Mosconi P. (1998). The Italian SF-36 Health Survey: translation, validation and norming. J. Clin. Epidemiol. 51, 1025–1036. 10.1016/S0895-4356(98)00094-8 9817120

[B4] Arocha Rodulfo J. I. (2019). Sedentary lifestyle a disease from xxi century. Clínica Investig. Arterioscler. 31, 233–240. 10.1016/j.arteri.2019.04.004 31221536

[B5] Aznar-Ballesta A. Peláez-Barrios E. M. Salas-Morillas A. Vernetta M. (2022). Motivation by, perceived quality of and satisfaction with sports services among young athletes: a psychological approach. Children 9, 1476. 10.3390/children9101476 36291412 PMC9601206

[B6] Baceviciene M. Jankauskiene R. Rutkauskaite R. (2023). The comparison of disordered eating, body image, sociocultural and coach-related pressures in athletes across age groups and groups of different weight sensitivity in sports. Nutrients 15, 2724. 10.3390/nu15122724 37375628 PMC10303047

[B7] Banack H. R. Stokes A. (2017). The ‘obesity paradox’ may not be a paradox at all. Int. J. Obes. 41, 1162–1163. 10.1038/ijo.2017.99 28584251

[B8] Bányai F. Zsila Á. Kökönyei G. Griffiths M. D. Demetrovics Z. Király O. (2021). The moderating role of coping mechanisms and being an e-sport player between psychiatric symptoms and gaming disorder: online survey. JMIR Ment. Health 8, e21115. 10.2196/21115 33755024 PMC8077919

[B9] Berengüí R. Angosto S. Hernández-Ruiz A. Rueda-Flores M. Castejón M. A. (2024). Body image and eating disorders in aesthetic sports: a systematic review of assessment and risk. Sci. Sports 39, 241–249. 10.1016/j.scispo.2023.03.006

[B10] Bjørlykhaug K. I. Karlsson B. Hesook S. K. Kleppe L. C. (2022). Social support and recovery from mental health problems: *a scoping review* . Nordic Soc. Work Res. 12, 666–697. 10.1080/2156857X.2020.1868553

[B11] Borowiec J. Banio-Krajnik A. Malchrowicz-Mośko E. Kantanista A. (2023). Eating disorder risk in adolescent and adult female athletes: the role of body satisfaction, sport type, BMI, level of competition, and training background. BMC Sports Sci. Med. Rehabil. 15, 91. 10.1186/s13102-023-00683-7 37491299 PMC10369723

[B12] Buckley G. Hall L. Lassemillante A.-C. Ackerman K. Belski R. (2019). Retired athletes and the intersection of food and body: a systematic literature review exploring compensatory behaviours and body change. Nutrients 11, 1395. 10.3390/nu11061395 31234314 PMC6627291

[B13] Budimir S. Probst T. Pieh C. (2021). Coping strategies and mental health during COVID-19 lockdown. J. Ment. Health 30, 156–163. 10.1080/09638237.2021.1875412 33502917

[B14] Bull F. C. Al-Ansari S. S. Biddle S. Borodulin K. Buman M. P. Cardon G. (2020). World Health Organization 2020 guidelines on physical activity and sedentary behaviour. Br. J. Sports Med. 54, 1451–1462. 10.1136/bjsports-2020-102955 33239350 PMC7719906

[B15] Büttner F. Howell D. R. Doherty C. Blake C. Ryan J. Delahunt E. (2021). Condition-specific health-related quality of life amongst amateur athletes six months and one-year following sport-related concussion: a prospective, follow-up. Phys. Ther. Sport 51, 71–78. 10.1016/j.ptsp.2021.06.011 34273667

[B16] Caricati L. Foà C. Fruggeri L. Tonarelli A. (2015). COPE-NVI-25: validazione italiana della versione ridotta della Coping Orientation to the Problems Experienced (COPE-NVI). Psicol. Della Salute, 123–140. 10.3280/PDS2015-002007

[B17] Chang C. Putukian M. Aerni G. Diamond A. Hong G. Ingram Y. (2020). Mental health issues and psychological factors in athletes: detection, management, effect on performance and prevention: American Medical Society for Sports Medicine Position Statement—executive Summary. Br. J. Sports Med. 54, 216–220. 10.1136/bjsports-2019-101583 31810972

[B18] Chieffi S. Castaldi C. Di Maio G. La Marra M. Messina A. Monda V. (2019). Attentional bias in the radial and vertical dimensions of space. Comptes Rendus Biol. 342, 97–100. 10.1016/j.crvi.2019.03.003 31036514

[B19] Chieffi S. Messina A. Villano I. Valenzano A. A. Nigro E. La Marra M. (2017). The use of velocity information in movement reproduction. Front. Psychol. 8, 983. 10.3389/fpsyg.2017.00983 28659849 PMC5466998

[B20] Chieffi S. Messina G. La Marra M. Iavarone A. Viggiano A. De Luca V. (2014). “Distractor interference in visual motor tasks,” in Horizons in neuroscience research, 151–160. Available online at: https://www.scopus.com/inward/record.uri?eid=2-s2.0-84953204618&partnerID=40&md5=f1ad7f440909329332fd386feb2a4fc8.

[B21] Childers D. K. Allison D. B. (2010). The ‘obesity paradox’: a parsimonious explanation for relations among obesity, mortality rate and aging? Int. J. Obes. 34, 1231–1238. 10.1038/ijo.2010.71 PMC3186057 20440298

[B22] Claussen M. C. Currie A. Koh Boon Yau E. Nishida M. Martínez V. Burger J. (2024). First international consensus statement on sports psychiatry. Scand. Med. Sci. Sports 34, e14627. 10.1111/sms.14627 PMC12810445 38610076

[B23] Crewther B. T. Kilduff L. P. Cook C. J. Cunningham D. J. Bunce P. J. Bracken R. M. (2012). Scaling strength and power for body mass differences in rugby union players. J. Sports Med. Phys. Fit. 52, 27–32. 22327083

[B24] Dahlstrand J. Friberg P. Fridolfsson J. Börjesson M. Arvidsson D. Ekblom Ö. (2021). The use of coping strategies “shift-persist” mediates associations between physical activity and mental health problems in adolescents: a cross-sectional study. BMC Public Health 21, 1104. 10.1186/s12889-021-11158-0 34107916 PMC8191033

[B25] Daley M. M. Reardon C. L. (2024). Mental health in the youth athlete. Clin. Sports Med. 43, 107–126. 10.1016/j.csm.2023.06.003 37949505

[B26] Dalkiliç M. (2017). The effect of cognitive flexibility of athletes on person-organization. IntJSCS 5, 213–224. 10.14486/IntJSCS674

[B27] Dišlere B. E. Mārtinsone K. Koļesņikova J. (2025). A scoping review of longitudinal studies of athlete burnout. Front. Psychol. 16, 1502174. 10.3389/fpsyg.2025.1502174 40028645 PMC11868109

[B28] Eather N. Wade L. Pankowiak A. Eime R. (2023). The impact of sports participation on mental health and social outcomes in adults: a systematic review and the ‘Mental Health through Sport’ conceptual model. Syst. Rev. 12, 102. 10.1186/s13643-023-02264-8 37344901 PMC10286465

[B29] Entwistle I. Francis P. Lees M. Hume P. Hind K. (2022). Lean mass, muscle strength, and muscle quality in retired rugby players: the UK rugby health Project. Int. J. Sports Med. 43, 958–963. 10.1055/a-1854-0052 35767990

[B30] Fatt S. J. George E. Hay P. Jeacocke N. Gotkiewicz E. Mitchison D. (2024). An umbrella review of body image concerns, disordered eating, and eating disorders in elite athletes. JCM 13, 4171. 10.3390/jcm13144171 39064211 PMC11278087

[B31] Filbay S. Pandya T. Thomas B. McKay C. Adams J. Arden N. (2019). Quality of life and life satisfaction in former athletes: a systematic review and meta-analysis. Sports Med. 49, 1723–1738. 10.1007/s40279-019-01163-0 31429036 PMC6789047

[B32] Forresi B. Michelini G. Sapuppo W. Costa G. Castellini G. Livellara S. (2022). Anger, personality traits and psychopathological symptoms in subjects exposed to negative interpersonal actions in workplaces: an observational study in a large sample attending a Center for Occupational Stress. Int. Arch. Occup. Environ. Health 95, 1763–1773. 10.1007/s00420-022-01868-2 35511292

[B33] Fruchart E. Rulence-Pâques P. (2022). Predicting sports performance from well-being: a mapping of professional athletes’, amateur athletes’ and non-athletes’ positions. Eur. Rev. Appl. Psychol. 72, 100793. 10.1016/j.erap.2022.100793

[B34] Fullerton D. J. Zhang L. M. Kleitman S. (2021). An integrative process model of resilience in an academic context: resilience resources, coping strategies, and positive adaptation. PLoS One 16, e0246000. 10.1371/journal.pone.0246000 33529232 PMC7853478

[B35] Furie K. Park A. L. Wong S. E. (2023). Mental health and involuntary retirement from sports post-musculoskeletal injury in adult athletes: a systematic review. Curr. Rev. Musculoskelet. Med. 16, 211–219. 10.1007/s12178-023-09830-6 37014610 PMC10188780

[B36] Fyfe J. J. Hamilton D. L. Daly R. M. (2022). Minimal-dose resistance training for improving muscle mass, strength, and function: a narrative review of current evidence and practical considerations. Sports Med. 52, 463–479. 10.1007/s40279-021-01605-8 34822137

[B37] Gao X. Zhong J. Li H. Pei Y. Li X. Zhang S. (2023). The relationship between perfectionism, self-perception of orofacial appearance, and mental health in college students. Front. Public Health 11, 1154413. 10.3389/fpubh.2023.1154413 37213631 PMC10196033

[B38] Gattino S. Rollero C. De Piccoli N. (2015). The influence of coping strategies on quality of life from a gender perspective. Appl. Res. Qual. Life 10, 689–701. 10.1007/s11482-014-9348-9

[B39] Geithner C. A. Lee A. M. Bracko M. R. (2006). Physical and performance differences among forwards, defensemen, and goalies in elite women’s ice Hockey. J. Strength Cond. Res. 20, 500–505. 10.1519/17375.1 16977704

[B40] González L. Castillo I. Balaguer I. (2019). Exploring the role of resilience and basic psychological needs as antecedents of enjoyment and boredom in female sports. Rev. Psicodidáctica English 24, 131–137. 10.1016/j.psicoe.2019.02.001

[B41] Graber R. Turner R. Madill A. (2016). Best friends and better coping: facilitating psychological resilience through boys’ and girls’ closest friendships. Br. J Psychol. 107, 338–358. 10.1111/bjop.12135 26110231

[B42] Grosprêtre S. Lepers R. (2016). Performance characteristics of Parkour practitioners: who are the traceurs? Eur. J. Sport Sci. 16, 526–535. 10.1080/17461391.2015.1060263 26267256

[B43] Habay J. Uylenbroeck R. Van Droogenbroeck R. De Wachter J. Proost M. Tassignon B. (2023). Interindividual variability in mental fatigue-related impairments in endurance performance: a systematic review and multiple meta-regression. Sports Med. - Open 9, 14. 10.1186/s40798-023-00559-7 36808018 PMC9941412

[B44] Hadebe N. F. Ramukumba T. S. (2020). Resilience and social support of young adults living with mental illness in the city of Tshwane, Gauteng province, South Africa. Curationis 43, e1–e7. 10.4102/curationis.v43i1.2084 PMC7756966 33354974

[B45] Harty P. S. Zabriskie H. A. Stecker R. A. Currier B. S. Moon J. M. Richmond S. R. (2021). Position-specific body composition values in female collegiate rugby union athletes. J. Strength Cond. Res. 35, 3158–3163. 10.1519/JSC.0000000000003314 31403573

[B46] Hopman W. M. Berger C. Joseph L. Barr S. I. Gao Y. Prior J. C. (2007). The association between body mass index and health-related quality of life: data from CaMos, a stratified population study. Qual. Life Res. 16, 1595–1603. 10.1007/s11136-007-9273-6 17957495

[B47] Huang Y. Su X. Si M. Xiao W. Wang H. Wang W. (2021). The impacts of coping style and perceived social support on the mental health of undergraduate students during the early phases of the COVID-19 pandemic in China: a multicenter survey. BMC Psychiatry 21, 530. 10.1186/s12888-021-03546-y 34706690 PMC8549419

[B48] Iavarone A. (2015). Memory for proprioceptive targets in bulimia nervosa. J. Psychiatry 18. 10.4172/Psychiatry.1000297

[B49] Ilardi C. R. Garofalo E. Chieffi S. Gamboz N. La Marra M. Iavarone A. (2020). Daily exposure to digital displays may affect the clock-drawing test: from psychometrics to serendipity. Neurol. Sci. 41, 3683–3690. 10.1007/s10072-020-04498-z 32506359

[B50] Ilardi C. R. Monda A. Iavarone A. Chieffi S. Casillo M. Messina A. (2024). The relationship between executive functions and body weight: sex as a moderating variable. Behav. Sci. 14, 258. 10.3390/bs14030258 38540561 PMC10967901

[B51] Jacobson J. Matthaeus L. (2014). Athletics and executive functioning: how athletic participation and sport type correlate with cognitive performance. Psychol. Sport Exerc. 15, 521–527. 10.1016/j.psychsport.2014.05.005

[B52] Jaekel J. (2024). The role of physical activity and fitness for children’s wellbeing and academic achievement. Pediatr. Res. 96, 1550–1551. 10.1038/s41390-024-03467-y 39122824 PMC11772221

[B53] Janiczak A. Devlin B. L. Forsyth A. Trakman G. L. (2022). A systematic review update of athletes’ nutrition knowledge and association with dietary intake. Br. J. Nutr. 128, 1156–1169. 10.1017/S0007114521004311 34706784

[B54] Kelly D. F. Chaloner C. Evans D. Mathews A. Cohan P. Wang C. (2014). Prevalence of pituitary hormone dysfunction, metabolic syndrome, and impaired quality of life in retired professional football players: a prospective study. J. Neurotrauma 31, 1161–1171. 10.1089/neu.2013.3212 24552537 PMC4082350

[B55] Kim S.-Y. Choi C. (2022). Differences in stress, stress-coping behavior, and quality of life based on the performance of Korean ladies professional golf association tour players. IJERPH 19, 6623. 10.3390/ijerph19116623 35682210 PMC9180033

[B56] Knettel B. A. Cherenack E. M. Rougier-Chapman C. Bianchi-Rossi C. (2023). Examining associations of coping strategies with stress, alcohol, and substance use among college athletes: implications for improving athlete coping. JIS 16, 186–204. 10.17161/jis.v16i2.18397

[B57] Koc Y. (2017). The effect of “physical education and sport culture” course on the attitudes of preservice classroom teachers towards physical education and sports. IJHE 6, 200. 10.5430/ijhe.v6n4p200

[B58] Koch P. Krenn B. (2021). Executive functions in elite athletes – comparing open-skill and closed-skill sports and considering the role of athletes’ past involvement in both sport categories. Psychol. Sport Exerc. 55, 101925. 10.1016/j.psychsport.2021.101925

[B59] Koelmel E. Hughes A. J. Alschuler K. N. Ehde D. M. (2017). Resilience mediates the longitudinal relationships between social support and mental health outcomes in multiple sclerosis. Archives Phys. Med. Rehabilitation 98, 1139–1148. 10.1016/j.apmr.2016.09.127 27789238

[B60] Krenn B. Finkenzeller T. Würth S. Amesberger G. (2018). Sport type determines differences in executive functions in elite athletes. Psychol. Sport Exerc. 38, 72–79. 10.1016/j.psychsport.2018.06.002

[B61] Krupa-Kotara K. Markowski J. Gdańska A. Grajek M. Działach E. Szlachta G. (2023). Global self-esteem, body composition, and physical activity in polish university students. Nutrients 15, 3907. 10.3390/nu15183907 37764691 PMC10536466

[B62] Kucharski B. Strating M. A. Ahluwalia Cameron A. Pascual-Leone A. (2018). Complexity of emotion regulation strategies in changing contexts: a study of varsity athletes. J. Contextual Behav. Sci. 10, 85–91. 10.1016/j.jcbs.2018.09.002

[B63] La Marra M. Ilardi C. R. Villano I. Carosella M. Staiano M. Iavarone A. (2022a). Functional relationship between inhibitory control, cognitive flexibility, psychomotor speed and obesity. Brain Sci. 12, 1080. 10.3390/brainsci12081080 36009143 PMC9405914

[B64] La Marra M. Messina A. Ilardi C. R. Staiano M. Di Maio G. Messina G. (2022b). Factorial model of obese adolescents: the role of body image concerns and selective depersonalization—a pilot study. IJERPH 19, 11501. 10.3390/ijerph191811501 36141782 PMC9517425

[B65] La Marra M. Messina A. Ilardi C. R. Verde G. Amato R. Esposito N. (2022c). The neglected factor in the relationship between executive functioning and obesity: the role of motor control. Healthcare 10, 1775. 10.3390/healthcare10091775 36141387 PMC9498752

[B66] La Marra M. Monda A. Monda M. Villano I. Chieffi S. Ricci M. (2024). Transcranial magnetic stimulation: a New possibility in obesity treatment. TONEUJ 18, e1874205X309047. 10.2174/011874205X309047240503104533

[B67] La Marra M. Villano I. Ilardi C. R. Carosella M. Staiano M. Iavarone A. (2022d). Executive functions in overweight and obese treatment-seeking patients: cross-sectional data and longitudinal perspectives. Brain Sci. 12, 777. 10.3390/brainsci12060777 35741662 PMC9220982

[B68] Larkin P. Carlon T. Sortino B. Greer S. Cuttiford T. Wijekulasuriya G. (2023). Anthropometry and physical performance in 13-year-old Australian talent-identified male and female athletes compared to an age-matched general population cohort. Children 10, 212. 10.3390/children10020212 36832341 PMC9954631

[B69] Lautenbach F. Leisterer S. Walter N. Kronenberg L. Manges T. Leis O. (2021). Amateur and recreational athletes’ motivation to exercise, stress, and coping during the corona crisis. Front. Psychol. 11, 611658. 10.3389/fpsyg.2020.611658 33584445 PMC7873522

[B70] Lavie C. J. Ozemek C. Carbone S. Katzmarzyk P. T. Blair S. N. (2019). Sedentary behavior, exercise, and cardiovascular health. Circulation Res. 124, 799–815. 10.1161/CIRCRESAHA.118.312669 30817262

[B71] Lerdal A. Andenæs R. Bjørnsborg E. Bonsaksen T. Borge L. Christiansen B. (2011). Personal factors associated with health-related quality of life in persons with morbid obesity on treatment waiting lists in Norway. Qual. Life Res. 20, 1187–1196. 10.1007/s11136-011-9865-z 21336658 PMC3178016

[B72] Le Roux J. Anema F. Janse Van Rensburg D. C. Kerkhoffs G. Gouttebarge V. (2023). Health conditions among retired elite rugby players: a scoping review. BMJ Open Sport Exerc Med. 9, e001573. 10.1136/bmjsem-2023-001573 PMC10401243 37547127

[B73] Limone P. Moscatelli F. Scarinci A. Carotenuto M. Messina A. Monda M. (2024). Brain neuromodulation effects on sport and nutrition: a narrative review. Teor. Metod. Fiz. Vihov. 24, 136–147. 10.17309/tmfv.2024.1.17

[B74] Liu C. Liang X. Sit C. H. P. (2024). Physical activity and mental health in children and adolescents with neurodevelopmental disorders: a systematic review and meta-analysis. JAMA Pediatr. 178, 247–257. 10.1001/jamapediatrics.2023.6251 38285440 PMC10825789

[B75] Liu M. Liu H. Qin Z. Tao Y. Ye W. Liu R. (2024). Effects of physical activity on depression, anxiety, and stress in college students: the chain-based mediating role of psychological resilience and coping styles. Front. Psychol. 15, 1396795. 10.3389/fpsyg.2024.1396795 38911957 PMC11191160

[B76] Logan N. E. Henry D. A. Hillman C. H. Kramer A. F. (2023). Trained athletes and cognitive function: a systematic review and meta-analysis. Int. J. Sport Exerc. Psychol. 21, 725–749. 10.1080/1612197X.2022.2084764

[B77] Mahindru A. Patil P. Agrawal V. (2023). Role of physical activity on mental health and well-being: a review. Cureus 15, e33475. 10.7759/cureus.33475 36756008 PMC9902068

[B78] Martin E. Beckham G. (2020). Force production during the sustained phase of Rugby scrums: a systematic literature review. BMC Sports Sci. Med. Rehabil. 12, 33. 10.1186/s13102-020-00174-z 32509309 PMC7249338

[B79] Martín-Rodríguez A. Belinchón-deMiguel P. Rubio-Zarapuz A. Tornero-Aguilera J. Martínez-Guardado I. Villanueva-Tobaldo C. (2024). Advances in understanding the interplay between dietary practices, body composition, and sports performance in athletes. Nutrients 16, 571. 10.3390/nu16040571 38398895 PMC10892519

[B80] Martinsen E. W. (2000). Physical activity for mental health. Tidsskr. Nor. Laegeforen 120, 3054–3056. 11109397

[B81] Martín-Talavera L. Gavín-Chocano Ó. Sanz-Junoy G. Molero D. (2023). Self-concept and self-esteem, determinants of greater life satisfaction in mountain and climbing technicians and athletes. EJIHPE 13, 1188–1201. 10.3390/ejihpe13070088 37504479 PMC10378547

[B82] Mason T. B. Crosby R. D. Kolotkin R. L. Grilo C. M. Mitchell J. E. Wonderlich S. A. (2017). Correlates of weight-related quality of life among individuals with binge eating disorder before and after cognitive behavioral therapy. Eat. Behav. 27, 1–6. 10.1016/j.eatbeh.2017.08.001 28843136 PMC5700842

[B83] McKeon G. Curtis J. Rosenbaum S. (2022). Promoting physical activity for mental health: an updated evidence review and practical guide. Curr. Opin. Psychiatry 35, 270–276. 10.1097/YCO.0000000000000796 35674711

[B84] McLoughlin E. Arnold R. Moore L. J. Slavich G. M. Fletcher D. (2024). A qualitative exploration of how lifetime stressor exposure influences sport performers’ health, well-being, and performance. Anxiety, Stress, and Coping 37, 233–250. 10.1080/10615806.2023.2246023 37665577 PMC11216060

[B85] McNeil D. G. Phillips W. J. Scoggin S. A. (2024). Examining the importance of athletic mindset profiles for level of sport performance and coping. Int. J. Sport Exerc. Psychol. 22, 995–1011. 10.1080/1612197X.2023.2180073

[B86] Menargues-Ramírez R. Sospedra I. Holway F. Hurtado-Sánchez J. A. Martínez-Sanz J. M. (2022). Evaluation of body composition in CrossFit® athletes and the relation with their results in official training. IJERPH 19, 11003. 10.3390/ijerph191711003 36078716 PMC9518485

[B87] Michaels C. Holman A. Teramoto M. Bellendir T. Krautgasser-Tolman S. Willick S. E. (2023). Descriptive analysis of mental and physical wellness in collegiate dancers. J. Dance Med. and Sci. 27, 173–179. 10.1177/1089313X231178091 37264604 PMC10456971

[B88] Michalsik L. B. Madsen K. Aagaard P. (2015). Technical match characteristics and influence of body anthropometry on playing performance in male elite team handball. J. Strength Cond. Res. 29, 416–428. 10.1519/JSC.0000000000000595 24978832

[B89] Milanović L. Živković D. Đošić A. Mitić P. Cicović B. Purenović-Ivanović T. (2022). BMI, body image, and quality of life—moderating role of physical activity. Appl. Sci. 12, 7061. 10.3390/app12147061

[B90] Miller T. W. Coates J. Plateau C. R. Barker J. B. (2024). Exploring the barriers and facilitators to mental health help-seeking behaviours in British elite track and field athletes. J. Appl. Sport Psychol. 36, 98–118. 10.1080/10413200.2023.2197962

[B91] Monda A. de Stefano M. I. Villano I. Allocca S. Casillo M. Messina A. (2024). Ultra-processed food intake and increased risk of obesity: a narrative review. Foods 13, 2627. 10.3390/foods13162627 39200554 PMC11353718

[B92] Monda V. La Marra M. Perrella R. Caviglia G. Iavarone A. Chieffi S. (2017). Obesity and brain illness: from cognitive and psychological evidences to obesity paradox. DMSO 10, 473–479. 10.2147/DMSO.S148392 PMC5701608 29200883

[B93] Moon I. Han J. (2022). Moderating effects of physical activity on the relationship between adverse childhood experiences and health-related quality of life. IJERPH 19, 668. 10.3390/ijerph19020668 35055490 PMC8775782

[B94] Murray M. Beattie J. McLeod C. Pedler D. Brumby S. Gabbe B. (2019). “It could have been a lot worse”: the psychological effects of farm-related serious injury in Victoria. Rural. Remote Health 19, 5323. 10.22605/RRH5323 31522511

[B95] Nippert A. H. Smith A. M. (2008). Psychologic stress related to injury and impact on sport performance. Phys. Med. Rehabilitation Clin. N. Am. 19, 399–418. 10.1016/j.pmr.2007.12.003 18395654

[B96] Nuetzel B. (2023). Coping strategies for handling stress and providing mental health in elite athletes: a systematic review. Front. Sports Act. Living 5, 1265783. 10.3389/fspor.2023.1265783 38033656 PMC10687549

[B97] Orbach I. Gutin H. Hoffman N. Blumenstein B. (2021). Motivation in competitive sport among female youth athletes. PSYCH 12, 943–958. 10.4236/psych.2021.126057

[B98] Paixão C. Oliveira S. Ferreira C. (2021). A comprehensive model of disordered eating among aesthetic athletic girls: exploring the role of body image-related cognitive fusion and perfectionistic self-presentation. Curr. Psychol. 40, 5727–5734. 10.1007/s12144-020-01142-z 34518749 PMC8426111

[B99] Paunescu A.-C. Préau M. Delpierre C. Jacob G. Pannard M. Delrieu L. (2024). Quality of life among French breast cancer survivors in comparison with cancer-free women: the Seintinelles study. BMC Women’s Health 24, 17. 10.1186/s12905-023-02827-w 38172846 PMC10765881

[B100] Pearce M. Garcia L. Abbas A. Strain T. Schuch F. B. Golubic R. (2022). Association between physical activity and risk of depression: a systematic review and meta-analysis. JAMA Psychiatry 79, 550–559. 10.1001/jamapsychiatry.2022.0609 35416941 PMC9008579

[B101] Pena-Pérez A. Portela-Pino I. (2023). Health and wellbeing among retired elite athletes: empirical evidence. jhse 18. 10.14198/jhse.2023.184.14

[B102] Penttilä E. Vuorinen V.-P. Kivimäki M. Ahlberg J. Airaksinen O. Tuomilehto H. (2022). Comparison of sleep between youth elite amateur athletes and professional athletes. Sport Sci. Health 18, 107–113. 10.1007/s11332-021-00780-5

[B103] Pfeifer C. E. Sacko R. S. Ortaglia A. Monsma E. V. Beattie P. F. Goins J. (2022). Fit to play? Health-related fitness levels of youth athletes: a pilot study. J. Strength Cond. Res. 36, 245–251. 10.1519/JSC.0000000000003430 31809462

[B104] Poitras V. J. Gray C. E. Borghese M. M. Carson V. Chaput J.-P. Janssen I. (2016). Systematic review of the relationships between objectively measured physical activity and health indicators in school-aged children and youth. Appl. Physiol. Nutr. Metab. 41, S197–S239. 10.1139/apnm-2015-0663 27306431

[B105] Prior E. Papathomas A. Rhind D. (2024). A systematic scoping review of athlete mental health within competitive sport: interventions, recommendations, and policy. Int. Rev. Sport Exerc. Psychol. 17, 903–925. 10.1080/1750984X.2022.2095659

[B106] Quesada O. Lauzon M. Buttle R. Wei J. Suppogu N. Kelsey S. F. (2022). Body weight and physical fitness in women with ischaemic heart disease: does physical fitness contribute to our understanding of the obesity paradox in women? Eur. J. Prev. Cardiol. 29, 1608–1614. 10.1093/eurjpc/zwac046 35244151 PMC9440958

[B107] Rebar A. L. Stanton R. Geard D. Short C. Duncan M. J. Vandelanotte C. (2015). A meta-meta-analysis of the effect of physical activity on depression and anxiety in non-clinical adult populations. Health Psychol. Rev. 9, 366–378. 10.1080/17437199.2015.1022901 25739893

[B108] Reinebo G. Alfonsson S. Jansson-Fröjmark M. Rozental A. Lundgren T. (2024). Effects of psychological interventions to enhance athletic performance: a systematic review and meta-analysis. Sports Med. 54, 347–373. 10.1007/s40279-023-01931-z 37812334 PMC10933186

[B109] Rogers D. L. Tanaka M. J. Cosgarea A. J. Ginsburg R. D. Dreher G. M. (2024). How mental health affects injury risk and outcomes in athletes. Sports Health A Multidiscip. Approach 16, 222–229. 10.1177/19417381231179678 PMC10916780 37326145

[B110] Rose S. Burton D. Kercher V. Grindley E. Richardson C. (2023). Enduring stress: a quantitative analysis on coping profiles and sport well-being in amateur endurance athletes. Psychol. Sport Exerc. 65, 102365. 10.1016/j.psychsport.2022.102365 37665837

[B111] Ruggiero G. M. Bassanini A. Benzi M. C. Boccalari F. Caletti E. Caselli G. (2017). Irrational and metacognitive beliefs mediate the relationship between content beliefs and gad symptoms: a study on a normal population. J. Rat-Emo Cognitive-Behav Ther. 35, 240–253. 10.1007/s10942-016-0253-z

[B112] Ruiz-Turrero J. Massar K. Kwasnicka D. Ten Hoor G. A. (2022). The relationship between compulsive exercise, self-esteem, body image and body satisfaction in women: a cross-sectional study. IJERPH 19, 1857. 10.3390/ijerph19031857 35162878 PMC8835063

[B113] Runacres A. Marshall Z. A. (2024). Prevalence of anxiety and depression in former elite athletes: a systematic review and meta-analysis. BMJ Open Sport Exerc Med. 10, e001867. 10.1136/bmjsem-2023-001867 PMC11667483 39720148

[B114] Ruscello B. Morganti G. De Fano A. Mancina F. Lunetta L. Di Mauro G. (2024). Comparing the anthropometrics, body composition, and strength performance of male and female Italian breaking athletes: a pilot study. Sports 12, 197. 10.3390/sports12070197 39058088 PMC11280681

[B115] Sánchez-Alcalá M. Aibar-Almazán A. Afanador-Restrepo D. F. Carcelén-Fraile M. D. C. Achalandabaso-Ochoa A. Castellote-Caballero Y. (2023). The impact of rhythmic physical activity on mental health and quality of life in older adults with and without cognitive impairment: a systematic review and meta-analysis. JCM 12, 7084. 10.3390/jcm12227084 38002696 PMC10672098

[B116] Sandi S. Syahara S. Firdaus K. Donie D. Rahman D. Zarya F. (2024). The role of nutritional status in improving physical endurance in athletes: a literature review. J. Info Kesehat. 22, 451–461. 10.31965/infokes.Vol22.Iss2.1557

[B117] Sanz-Matesanz M. Martínez-Aranda L. M. Gea-García G. M. (2024). Effects of a physical training program on cognitive and physical performance and health-related variables in professional esports players: a pilot study. Appl. Sci. 14, 2845. 10.3390/app14072845

[B118] Sapuppo W. Giacconi D. Monda V. Messina A. Allocca S. Chieffi S. (2024a). Functional characteristics and coping strategies among rugby athletes: a cluster analysis approach. JPM 14, 292. 10.3390/jpm14030292 38541034 PMC10970941

[B119] Sapuppo W. Monda A. Giacconi D. Gregori Grgič R. Saccenti D. Mineo C. M. (2024b). Health-related quality of life in rugby athletes: the role of dietary supplements and their consumption. Sports 12, 270. 10.3390/sports12100270 39453236 PMC11511494

[B120] Sapuppo W. Sbrizzi C. Boltri M. La Marra M. Giacconi D. Saccenti D. (2024c). Assessment tools for clinical excoriation (skin picking) disorder: a mini review for diagnosing and monitoring symptoms severity. Curr. Psychol. 43, 26134–26143. 10.1007/s12144-024-06300-1

[B121] Sarkar M. Fletcher D. (2014). Psychological resilience in sport performers: a review of stressors and protective factors. J. Sports Sci. 32, 1419–1434. 10.1080/02640414.2014.901551 24716648

[B122] Sbrizzi C. Sapuppo W. (2021). Effects of pet therapy in elderly patients with neurocognitive disorders: a brief review. Dement. Geriatr. Cogn. Disord. Extra 11, 198–206. 10.1159/000518469 PMC8460886 34703453

[B123] Scharfen H. Memmert D. (2019). Measurement of cognitive functions in experts and elite athletes: a meta‐analytic review. Appl. Cogn. Psychol. 33, 843–860. 10.1002/acp.3526

[B124] Schinke R. J. Henriksen K. Moore Z. E. Stambulova N. Bartley J. Cosh S. (2024). International society of sport psychology position stand: elite athlete mental health revisited. Int. J. Sport Exerc. Psychol. 22, 775–801. 10.1080/1612197X.2024.2359872

[B125] Schuch F. B. Vancampfort D. (2021). Physical activity, exercise, and mental disorders: it is time to move on. Trends Psychiatry Psychother. 43, 177–184. 10.47626/2237-6089-2021-0237 33890431 PMC8638711

[B126] Schut M. Schut H. (1999). The dual process model of coping with bereavement: rationale and description. Death Stud. 23, 197–224. 10.1080/074811899201046 10848151

[B127] Sheehan R. B. Herring M. P. Campbell M. J. (2018). Associations between motivation and mental health in sport: a test of the hierarchical model of intrinsic and extrinsic motivation. Front. Psychol. 9, 707. 10.3389/fpsyg.2018.00707 29867672 PMC5953338

[B128] Silva A. M. Nunes C. L. Jesus F. Francisco R. Matias C. N. Cardoso M. (2022). Effectiveness of a lifestyle weight-loss intervention targeting inactive former elite athletes: the Champ4Life randomised controlled trial. Br. J. Sports Med. 56, 394–401. 10.1136/bjsports-2021-104212 34598935

[B129] Silva D. A. S. Petroski E. L. Gaya A. C. A. (2013). Anthropometric and physical fitness differences among Brazilian adolescents who practise different team court sports. J. Hum. Kinet. 36, 77–86. 10.2478/hukin-2013-0008 23717357 PMC3661897

[B130] Simati S. Kokkinos A. Dalamaga M. Argyrakopoulou G. (2023). Obesity paradox: fact or fiction? Curr. Obes. Rep. 12, 75–85. 10.1007/s13679-023-00497-1 36808566

[B131] Singh B. Olds T. Curtis R. Dumuid D. Virgara R. Watson A. (2023). Effectiveness of physical activity interventions for improving depression, anxiety and distress: an overview of systematic reviews. Br. J. Sports Med. 57, 1203–1209. 10.1136/bjsports-2022-106195 36796860 PMC10579187

[B132] Sparks J. R. Wang X. Lavie C. J. Sui X. (2024). Physical activity, cardiorespiratory fitness, and the obesity paradox with consideration for racial and/or ethnic differences: a broad review and call to action. Rev. Cardiovasc. Med. 25, 291. 10.31083/j.rcm2508291 39228496 PMC11366992

[B133] Staśkiewicz-Bartecka W. Grochowska-Niedworok E. Zydek G. Grajek M. Kiciak A. Białek-Dratwa A. (2023). Anthropometric profiling and changes in segmental body composition of professional football players in relation to age over the training macrocycle. Sports 11, 172. 10.3390/sports11090172 37755849 PMC10537170

[B134] Stefanovics E. A. Potenza M. N. Tsai J. (2024). Obesity in U.S. low-income veterans:Prevalence, clinical characteristics, and homelessness. J. Psychiatric Res. 173, 317–325. 10.1016/j.jpsychires.2024.03.041 38574595

[B135] Street J. H. Boos Z. P. Fial A. Lennon S. L. Smith C. S. Creasy S. A. (2023). Long-term function, body composition and cardiometabolic health in midlife former athletes: a scoping review. BMJ Open Sport Exerc Med. 9, e001605. 10.1136/bmjsem-2023-001605 PMC10619025 37920279

[B136] Taheri M. Irandoust K. Reynoso-Sánchez L. F. Muñoz-Helú H. Cruz-Morales K. N. Torres-Ramírez R. (2023). Effects of home confinement on physical activity, nutrition, and sleep quality during the COVID-19 outbreak in amateur and elite athletes. Front. Nutr. 10, 1143340. 10.3389/fnut.2023.1143340 37139442 PMC10150803

[B137] Tomaszek K. Muchacka-Cymerman A. (2019). Sex differences in the relationship between student school burnout and problematic internet use among adolescents. IJERPH 16, 4107. 10.3390/ijerph16214107 31653105 PMC6862502

[B138] Torrente-Sánchez M. J. Ferrer-Márquez M. Estébanez-Ferrero B. Jiménez-Lasserrotte M. D. M. Ruiz-Muelle A. Ventura-Miranda M. I. (2021). Social support for people with morbid obesity in a bariatric surgery programme: a qualitative descriptive study. IJERPH 18, 6530. 10.3390/ijerph18126530 34204427 PMC8297395

[B139] Toselli S. Rinaldo N. Mauro M. Grigoletto A. Zaccagni L. (2022). Body image perception in adolescents: the role of sports practice and sex. IJERPH 19, 15119. 10.3390/ijerph192215119 36429834 PMC9690021

[B140] Trajković N. Mitić P. M. Barić R. Bogataj Š. (2023). Editorial: effects of physical activity on psychological well-being. Front. Psychol. 14, 1121976. 10.3389/fpsyg.2023.1121976 36743250 PMC9890147

[B141] Uchino B. N. (2006). Social support and health: a review of physiological processes potentially underlying links to disease outcomes. J. Behav. Med. 29, 377–387. 10.1007/s10865-006-9056-5 16758315

[B142] Van Baak M. A. Pramono A. Battista F. Beaulieu K. Blundell J. E. Busetto L. (2021). Effect of different types of regular exercise on physical fitness in adults with overweight or obesity: systematic review and meta‐analyses. Obes. Rev. 22, e13239. 10.1111/obr.13239 33939229 PMC8365680

[B143] Varghese M. Ruparell S. LaBella C. (2022). Youth athlete development models: a narrative review. Sports Health A Multidiscip. Approach 14, 20–29. 10.1177/19417381211055396 PMC8669922 34758649

[B144] Vasileva F. Shukova-Stojmanovska D. Vasilev A. Georgiev G. (2022). BMI and nutritional status in physical active population involved in recreational sport. J. Anthr. Sport Phis. Educ. 6, 13–19. 10.26773/jaspe.220103

[B145] Villano I. Ilardi C. R. Arena S. Scuotto C. Gleijeses M. G. Messina G. (2021a). Obese subjects without eating disorders experience binge episodes also independently of emotional eating and personality traits among university students of southern Italy. Brain Sci. 11, 1145. 10.3390/brainsci11091145 34573166 PMC8465169

[B146] Villano I. La Marra M. Allocca S. Ilardi C. R. Polito R. Porro C. (2022). The role of nutraceutical supplements, monacolin K and astaxanthin, and diet in blood cholesterol homeostasis in patients with myopathy. Biomolecules 12, 1118. 10.3390/biom12081118 36009012 PMC9405860

[B147] Villano I. La Marra M. Messina A. Di Maio G. Moscatelli F. Chieffi S. (2021b). Effects of vegetarian and vegan nutrition on body composition in competitive futsal athletes. Prog. Nutr. 23, e2021126. 10.23751/pn.v23i2.11366

[B148] Voorheis P. Silver M. Consonni J. (2023). Adaptation to life after sport for retired athletes: a scoping review of existing reviews and programs. PLoS ONE 18, e0291683. 10.1371/journal.pone.0291683 37733723 PMC10513329

[B149] Walsh J. Heazlewood I. T. Climstein M. (2018). Body mass index in master athletes: review of the literature. J. Lifestyle Med. 8, 79–98. 10.15280/jlm.2018.8.2.79 30474004 PMC6239137

[B150] Warburton D. E. R. Nicol C. W. Bredin S. S. D. (2006). Health benefits of physical activity: the evidence. Can. Med. Assoc. J. 174, 801–809. 10.1503/cmaj.051351 16534088 PMC1402378

[B151] Wong Y. Qiu H. Turner S. (2024). Athlete experiences of transitioning from amateur to professional sports: a grounded theory approach. Int. J. Sport Stud. Health 7, 62–70. 10.61838/kman.intjssh.7.3.9

[B152] Yang P. Xu R. Le Y. (2024). Factors influencing sports performance: a multi-dimensional analysis of coaching quality, athlete well-being, training intensity, and nutrition with self-efficacy mediation and cultural values moderation. Heliyon 10, e36646. 10.1016/j.heliyon.2024.e36646 39263094 PMC11386267

[B153] Yongtawee A. Park J. Kim Y. Woo M. (2022). Athletes have different dominant cognitive functions depending on type of sport. Int. J. Sport Exerc. Psychol. 20, 1–15. 10.1080/1612197X.2021.1956570

[B154] Zaccagni L. Gualdi-Russo E. (2023). The impact of sports involvement on body image perception and ideals: a systematic review and meta-analysis. IJERPH 20, 5228. 10.3390/ijerph20065228 36982136 PMC10049477

[B155] Zhao J. Li X. Liang C. Yan Y. (2025). Can exercise-mediated adipose browning provide an alternative explanation for the obesity paradox? IJMS 26, 1790. 10.3390/ijms26051790 40076419 PMC11898606

[B156] Zwartkruis V. W. Suthahar N. Idema D. L. Mahmoud B. Van Deutekom C. Rutten F. H. (2023). Relative fat mass and prediction of incident atrial fibrillation, heart failure and coronary artery disease in the general population. Int. J. Obes. 47, 1256–1262. 10.1038/s41366-023-01380-8 37684330

